# Transcription factor SNAI2 exerts pro-tumorigenic effects on glioma stem cells via PHLPP2-mediated Akt pathway

**DOI:** 10.1038/s41419-021-04481-2

**Published:** 2022-06-02

**Authors:** Lilei Peng, Jie Fu, Yitian Chen, Yang Ming, Haiping He, Shan Zeng, Chuanhong Zhong, Ligang Chen

**Affiliations:** 1 Department of Neurosurgery, The Affiliated Hospital of Southwest Medical University, Luzhou, 646000 PR China; 2 Sichuan Clinical Research Center of Neurosurgery, Luzhou, 646000 PR China; 3 Academician (Expert) Workstation of Sichuan Province, Luzhou, 646000 PR China; 4 Neurological Diseases and Brain Function Laboratory, The Affiliated Hospital of Southwest Medical University, Luzhou, 646000 PR China; 5 Department of Neurology, The Affiliated Hospital of Southwest Medical University, Luzhou, 646000 PR China; 6 Department of Clinical Medicine, Medical College of Soochow University, Suzhou, 215123 PR China

**Keywords:** Stem cells, Cancer stem cells

## Abstract

The current study aimed to investigate the effects associated with SNAI2 on the proliferation of glioma stem cells (GSCs) to elucidate its underlying molecular mechanism in the development of glioma. The expression of Snail family transcriptional repressor 2 (SNAI2) in glioma tissues was initially predicted via bioinformatics analysis and subsequently confirmed by reverse transcription quantitative polymerase chain reaction (RT-qPCR), which revealed that SNAI2 was highly expressed in glioma tissues as well as GSCs, with an inverse correlation with overall glioma patient survival detected. Loss- and gain- of-function assays were performed to determine the roles of SNAI2 and pleckstrin homology domain and leucine rich repeat protein phosphatase 2 (PHLPP2) on GSC viability, proliferation and apoptosis. Data were obtained indicating that SNAI2 promoted the proliferation of GSCs, while overexpressed PHLPP2 brought about a contrasting trend. As detected by chromatin immunoprecipitation, RT-qPCR and agarose gel electrophoresis, SNAI2 bound to the promoter region of PHLPP2 and repressed the transcription of PHLPP2 while SNAI2 was found to inhibit PHLPP2 resulting in activation of the Akt pathway. Finally, the roles of SNAI2 and PHLPP2 were verified in glioma growth in nude mice xenografted with tumor. Taken together, the key findings of the present study suggest that SNAI2 may promote the proliferation of GSCs through activation of the Akt pathway by downregulating PHLPP2.

## Introduction

Glioma is a primary intracranial tumor with various histological subtypes, including astrocytomas, glioblastoma multiform, oligodendrogliomas as well as mixed tumors [1]. Malignant glioma is often characterized by its pervasive infiltration into nearby brain parenchyma [2]. Glioma often manifests itself with a distinct degree of invasiveness and heterogeneity [3] and has been reported to account for approximately 30% of brain and central nervous system tumors [4]. Current existing therapy for malignant glioma is a combination of chemotherapy and radiotherapy [5]. Despite attempts to improve treatment protocols, glioma patients still suffer from a low 5-year survival rate, reported to be as low as 5.5%, with a median survival period of 14.5–16.6 months [6]. Glioma stem cells (GSCs) have been implicated in tumor regrowth of glioma which is often treatment-resistant [7]. Therefore, it is imperative that more effective treatment targets are uncovered capable of inhibiting GSC progression.

The Snail family transcriptional repressor 2 (SNAI2) has been highlighted as a zinc-finger transcription factor well regarded for its ability to inhibit the expression of encoding genes [8]. Intriguingly, a previous study emphasized SNAI2 as an oncogenic transcriptional repressor in the development of glioma [9]. Moreover, the upregulation of SNAI2 by zinc-fingers and homeoboxes 1 has been reported to accelerate the proliferation and invasion of glioblastoma cells [10]. Our bioinformatics prediction identified the existence of binding sites between SNAI2 and the promoter of pleckstrin homology domain and leucine rich repeat protein phosphatase 2 (PHLPP2). PHLPP has been previously documented as a type of serine/threonine phosphatase capable of modulating cell growth via dephosphorylation of the AGC family of kinases [11]. Existing literature has indicated that the expression of PHLPP2 is diminished in glioma [12] highlighting its promise as a treatment target for brainstem glioma [13]. Studies have suggested that PHLPP2 may inactivate protein kinase B (Akt) to suppress cell-cycle progression while facilitating cell apoptosis [14] As a serine/threonine kinase belonging to AGC kinases, Akt has been reported to influence crucial cellular biological functions [15]. Accumulating studies continue to highlight the stimulatory role of the Akt pathway in the progression of glioma [16, 17]. Notably, downregulated PHLPP2 by microRNA-93 (miR-93) has been reported to activate phosphoinositide 3-kinase (PI3K)/Akt signaling, which ultimately favors glioma cell promotion [18]. Based on the aforementioned studies as well as our bioinformatics finding, we proposed the hypothesis that SNAI2 participates in the regulation of GSC progression, with involvement of PHLPP2 as well as the Akt pathway.

## Materials and methods

### Study subjects

A total of 80 patients with glioma (45 males and 35 females; aged from 22 to 80 years, with an average age of 54.50 ± 13.36 years) who underwent surgical resection between June 2013 and June 2016 in The Affiliated Hospital of Southwest Medical University were enrolled into the study. All patients were yet to receive any anti-tumor treatment prior to surgery. All participants were deemed suitable for surgery based on their respective preoperative imaging results, with the postoperative pathological section diagnosis confirmed to be glioma. All patients with distant metastasis and cachexia were excluded from the study. Healthy volunteers were enrolled with matched age and gender with that of patients, at a ratio of 1:1. The clinical data of the patients were collected, with all patients followed up to understand the detailed situation and clinical outcomes following treatment. The follow-up period was from completion of surgery until June 2019, which lasted for 3–36 months.

### Cell treatment

GSC lines, namely, A172 (CRL-1620), and LN229 (CRL-2611), purchased from American Type Culture Collection (Manassas, VA, USA), and SHG-44 (TCHu 48, Cellbank, Shanghai, China) were used in the experiment. The cells were cultured with Dulbecco’s modified Eagle’s medium (A172, LN229) and Eagle’s minimal essential medium (SHG-44, Gibco, Carlsbad, CA, USA) containing 10% fetal bovine serum, 10 μg/ml streptomycin and 100 U/ml penicillin in an incubator at 37 °C with 5% CO_2_ (Thermo Fisher Scientific Inc., Waltham, MA, USA). Human astrocytes (#1800, ScienCell, San Diego, CA, USA) were cultured with astrocyte medium (#1801) and regarded as the control.

The cells were trypsinized upon exhibiting logarithmic growth, and seeded to a six-well plate. After regular culture for 24 h, the cells were transfected according to the instructions of Lipofectamine 2000 (Invitrogen, Carlsbad, CA, USA) upon reaching 60% confluence. The cells were transfected with negative control for small interfering RNA (siRNA) (si-NC) (5-GGGUGAACUCACGUCAGAA-3 sequence), SNAI2 overexpression plasmid (oe-SNAI2), SNAI2-knockdown (KD) (5-GACAGGTAUCUCTUCGUUAUC-3 sequence), NC for overexpression plasmid (oe-NC), and PHIPP2 overexpression plasmid (oe-PHIPP2). After 48 h of transfection, the transfection efficiency was confirmed via reverse transcription quantitative polymerase chain reaction (RT-qPCR). The expression plasmids were purchased from GenePharma Co. Ltd. (Shanghai, China) with the concentration of the plasmids in this experiment confirmed to be 50 ng/ml. pSilencer 4.1-CMV neo (G418 resistance) vector or Pegfp-4.1N (G418 resistance) served as the backbone for the aforementioned vectors/plasmids, and entrusted the construction of GenePharma.

### RT-qPCR

Trizol reagent (15596026, Invitrogen) was utilized for total RNA extraction purposes. In accordance with the instructions of PrimeScript RT Regent Kit (RR047a, Takara, Otsu, Shiga, Japan), the RNA was reversely transcribed into complementary DNA (cDNA). The synthesized cDNA was determined by RT-qPCR with a Fast SYBR Green PCR kit (Applied Biosystems, Inc., Foster City, CA, USA) and ABI PRISM 7300 RT-PCR system (Applied Biosystems). Three duplicates were set for each well. Glyceraldehyde-3-phosphate dehydrogenase (GAPDH) was employed as an internal parameter to analyze the relative expression of the target gene using the 2^−ΔΔ^Ct method. The primers are depicted in Supplementary Table 1.

### Western blot assay

The cells were trypsinized, collected, and lysed using an enhanced radioimmunoprecipitation assay lysate (Boster, Wuhan, China) containing protease inhibitors. A bicinchoninic acid protein quantitative kit (Boster) was used to determine the protein concentration. The protein was separated by sodium dodecyl sulfate-polyacrylamide gel electrophoresis, and subsequently transferred onto a polyvinylidene fluoride membrane and sealed with 5% bovine serum albumin (BSA) at room temperature for 2 h to block non-specific binding. Next, the membrane was incubated at 4 °C overnight with diluted primary rabbit antibodies to SNAI2 (9585, 1:1000), PHLPP2 (ab71973, 1:100), Akt (ab8805, 1:1000), phosphorylated (p)-Akt (T308; ab38449, 1:500), GAPDH (ab8227, 1:2000), phosphorylated mammalian target of rapamycin complexes (p-mTORC) (ab109268, 1:100), and mTORC (ab2732, 1:100). The next day, the membrane was washed and incubated with horseradish peroxidase (HRP) labeled goat anti-rabbit secondary antibody (ab181602; 1:2000) at room temperature for 1 h. The antibody to SNAI2 was purchased from Cell Signaling Technologies (Beverly, MA, USA), while the other antibodies were all purchased from Abcam Inc. (Cambridge, MA, USA). Enhanced chemiluminescence working solution (EMD Millipore, Billerica, Massachusetts, USA) was employed to incubate the membrane at room temperature for 1 min, followed by sealing of the membrane with plastic films, exposure with X-ray film in a dark box for 5–10 min, developed and fixed. ImageJ analysis software was used to quantify the gray level of each band in Western blot images, with GAPDH serving as the internal parameter.

### 5-ethynyl-2-deoxyuridine (EdU) assay

The cells were seeded into a 24-well plate, with three duplicates set for each cell treatment group. EdU was added into the culture medium to reach a concentration of 10 μmol/l, followed by incubation for 2 h. Following removal of the culture medium, the cells were fixed at room temperature with phosphate buffer saline (PBS) solution containing 4% paraformaldehyde for 15 min, and incubated with PBS containing 0.5% triton-100 for 20 min. Next, 100 μl of dye solution was added to each well, followed by incubation at room temperature for 30 min in the dark. Next, 4′,6-diamidino-2-phenylindole (DAPI) was applied to dye the nuclei for 5 min followed by sealing of the cells. Next, 6–10 visual fields were selected at random and observed under a fluorescence microscope (FM-600, Pudan Optical Instrument Co., Ltd., Shanghai, China). The number of positive cells in each visual field was recorded. EdU labeling rate (%) = the number of positive cells/(the number of positive cells + number of negative cells) × 100%.

### Flow cytometry

After cell treatment and counting, the cells were resuspended with PBS containing 2% BSA to reach a cell concentration of 1 × 10^6^ cells/ml. No antibody was added to the blank control tube, with 5–20 μl rabbit antibody to CD133 (ab19898, Abcam) subsequently added to the sample tubes, followed by mixing and incubation at room temperature for 30 min. The cells were centrifuged at 1000 rpm for 5 min, and subsequently resuspended with 100 μl PBS. Fluorescence-labeled secondary antibody, namely, goat anti-rabbit to immunoglobulin G (IgG) H&L (HRP) (ab205718, Abcam) was added to the cells and incubated at room temperature under dark conditions for 30 min. Following centrifugation at 1000 rpm for 5 min, the cells were resuspended with 100–200 μl PBS, with different samples examined using a flow cytometer (DxFLEX, Beckman Coulter Inc., Chaska, MN, USA).

As per the manual, cell apoptosis was detected by Annexin V-Fluorescein Isothiocyanate (FITC) kit (BD Bioscience, NY, USA). Briefly, cells were detached by trypsin, collected, washed with cold PBS and centrifuged following the culmination of a 3-day transfection. The cell precipitate was resuspended in cold binding buffer solution and added with Annexin V-FITC (1.25 μl/0.5 ml) and propidium iodide (10 μl/0.5 ml). Flow cytometer (DxFLEX, Beckman) was employed for detection purposes. Three duplicates were set for each well.

### Immunofluorescence

In the culture plate, cells were fixed with 4% paraformaldehyde for 15 min, immersed in PBS and treated with 0.5% Triton X-100 at room temperature for 20 min. Normal goat serum was added in a dropwise manner to the slides, which were then sealed at room temperature for 30 min. Next, diluted primary antibodies to CD133 (ab19898, Abcam) and Nestin (ab105389, Abcam) were added to each slide, followed by overnight incubation in a wet box at 4 °C. The following day, the fluorescent secondary antibody goat anti-rabbit IgG H&L (HRP) (ab205718, Abcam) was used to incubate the slides in a wet box at 20–37 °C for 1 h. The samples were stained with DAPI, followed by sealing with anti-fade agent, and subsequently observed and photographed under a fluorescence microscope (FM-600, Pudan Optical Instrument Co., Ltd., Shanghai, China).

### Spheroid formation assay

The GSCs were cultured in the medium containing 20 ng/ml basic fibroblast growth factor and epidermal growth factor, 5 μg/ml insulin (Sigma-Aldrich Chemical Company, St Louis, MO, USA), 0.4% BSA (Invitrogen) and 0.02% B27 (Invitrogen) and subcultured in a six-well plate. After 7 days of culture, the cells were fixed with 10% formalin and photographed under a normal microscope. The sphere-forming efficiency which characterizes the sphere-forming ability was subsequently calculated. The calculation formula of sphere-forming efficiency was: the number of cell spheroids per well/the total number of cells initially seeded per well.

### Chromatin immunoprecipitation (ChIP)

ChIP was performed using a ChIP kit (Millipore). Briefly, the cells were fixed with 1% formaldehyde at room temperature for 10 min upon reaching 70–80% confluence, with the DNA and protein cross-linked in the cells. After lysis, the complex was randomly broken into 500–1000 bp fragments post ultrasonic treatment. Following centrifugation at 4 °C and 13,000 rpm, the supernatant was collected and separately placed into different three tubes. The positive control antibody RNA polymerase II, the NC antibody to human IgG and 2 μg rabbit antibody to SNAI2 (ab27568, Abcam) were subsequently added to the tubes, respectively, followed by overnight incubation at 4 °C. The endogenous DNA-protein complex was precipitated with protein agarose/sepharose, followed by short centrifugation and removal of the supernatant. The complex was subjected to overnight de-crosslinking at 65 °C and the enrichment of SNAI2 in the promoter region of PHLPP2 was evaluated by RT-qPCR and agarose gel electrophoresis. The primers are depicted in Supplementary Table 2.

### Dual luciferase reporter gene assay

The GSCs (3 × 10^4^ cells/well) were seeded into a 24-well plate, and permitted to rest for 24 h. The cells were transfected with the aforementioned mentioned plasmids and 1.5 ng pRL-TK Renilla plasmids using Lipofectamine 3000 reagent (Thermo Fisher Scientific Inc.). After 48 h of transfection, the firefly and renilla luciferases were measured using dual luciferase assay kits (Promega Corporation, Madison, WI, USA).

### Xenograft in nude mice

Fifty healthy nude mice (6–8 weeks old) (Institute of Medicinal Biotechnology, Chinese Academy of Medical Sciences, Beijing, China). Each nude mouse was raised in separate cages in a specific-pathogen-free animal laboratory (humidity: 60–65%; temperature: 22–25 °C), permitted with free access to food and water under 12-h light/dark cycles. The experiment started one week after adaptive feeding. Before the experiment, the health status of nude mice was observed.

Ten nude mice were randomly used as control. GSCs (1 × 10^7^ cells/200 μl) that had been stably-transfected with oe-NC, oe-SNAI2, oe-PHLPP2, or oe-SNAI2 + oe-PHLPP2 were subcutaneously injected with into 1–2 cm under the armpit of the nude mice (*n* = 10). Tumor growth was monitored on a weekly basis by means of measuring the width (*W*) and length (*L*) with a caliper, with the volume (*V*) of the tumor was calculated using the formula *V* = (*W*^2^ × *L*)/2. Five weeks after injection, the mice were euthanized and the tumor tissues were removed out, weighed, photographed and a part of tissues were fixed with 4% paraformaldehyde solution. The remainder of the tumor tissues were frozen in liquid nitrogen and stored at −80 °C for subsequent experimentation.

### Hematoxylin-eosin (H&E) staining

The sections were rinsed in xylene I and II, each for 10 min, followed by anhydrous ethanol I and II each for 5 min, in gradient alcohol (95, 90, 80, and 70%), each for 5 min, and finally in distilled water. The sections were dyed with Harris hematoxylin for 3–8 min, and washed under tap water, followed by differentiation with 1% hydrochloric acid alcohol for several seconds, and treatment with 0.6% ammonia water to provide a blue gamut. After staining with eosin for 1–3 min, the sections were immersed in 95% alcohol I and II, and in anhydrous ethanol I and II, each for 5 min. Following dehydration and clearing with xylene I and II 5 min each), the sections were dried naturally and sealed with neutral gum, followed by microscopic observation, image acquisition and analysis. The nuclei were stained bright blue with hematoxylin, cartilage matrix and calcium salt granules dark blue, and mucus gray blue. Eosinophilic granules in the cytoplasm were stained bright red with strong reflection. Collagen fibers were light pink, elastic fibers appeared bright pink, red blood cells were orange-red, and protein liquid was in pink color.

### Immunohistochemistry

Tumor samples from both the nude mice as well as the patients were fixed in 4% paraformaldehyde and embedded in paraffin. The paraffin sections (5 μm in thickness) were prepared using conventional methods. More specifically, the sections were dewaxed in xylene, rehydrated with distilled water, and then subjected to antigen repair in 0.01 M citric acid buffer at 95 °C for 30 min. The sections were subsequently placed on slides and incubated overnight with rabbit antibodies to SNAI2 (9585, 1:100, Cell Signaling Technologies), PHLPP2 (ab71973, 1:100, Abcam), p-Akt (phospho T308) (ab38449, 1:500, Abcam), and p-mTORC (ab109268, 1:100, Abcam) at 4 °C. The slides were subsequently treated with diluted goat anti-rabbit to IgG (ab6795, Abcam), and the avidin-conjugated HRP and diaminobiphenylamine were employed as the substrates for color development purposes. Following counterstaining with hematoxylin, Japanese Nikon image analysis software was used for photographing.

The SNAI2 immunohistochemistry results were independently scored by two histopathologists based on staining intensity and percentage of positive tumor cells in a blind manner. The best cut-off value of SNAI2 expression was 6 as predicted by X-tile software. A score ≥6 suggested that SNAI2 was highly expressed; otherwise, SNAI2 was poorly expressed.

### Statistical analysis

Measurement data analysis was conducted using statistical software SPSS 21.0 (IBM Corp., Armonk, NY, USA) and expressed as the mean ± standard deviation. Unpaired *t*-tests were applied to compare data between two groups, while one-way analysis of variance (ANOVA) was used to compare data among multiple groups. Repeated measures ANOVA was employed to compare tumor volume at different time points. The Kaplan–Meier method was used to evaluate the relationship between the expression of SNAI2 and the overall survival (OS) of patients. Univariate analysis was performed using Log-rank test. Chi-square tests were performed to analyze the medical records. *p* < 0.05 was considered to be indicative of statistically significant difference.

## Results

### SNAI2 expression was upregulated in glioma tissues

**Fig. 1:**
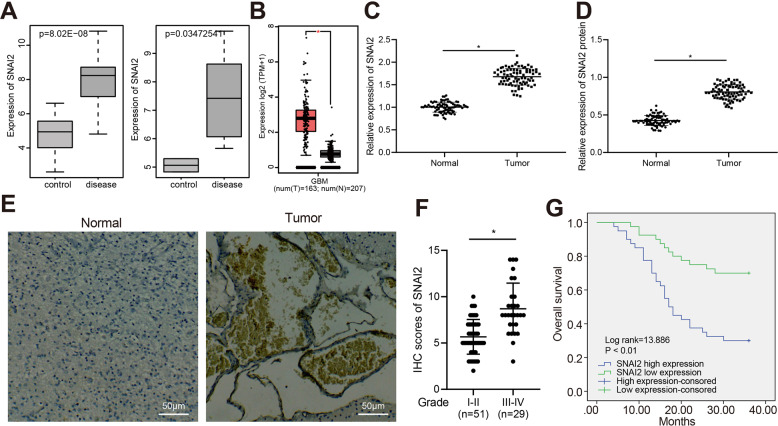
SNAI2 expression is upregulated in glioma tissues. **A** The high expression of SNAI2 in glioma as predicted on the GSE50161 (left panel) and GSE15824 dataset (right panel). In the figure, the *x*-axis indicates the sample type, the *y*-axis indicates the gene expression value, and the upper left indicates the differential *p* value. **B** The expression of SNAI2 gene in TCGA glioma dataset. The *x*-axis indicates the sample type, the *y*-axis indicates the expression value, the red box plot indicates the tumor sample, and the gray indicates normal sample. *p < 0.05 vs. normal tissues. **C** SNAI2 mRNA expression in glioma tissues and adjacent normal tissues as determined by RT-qPCR. *n* = 80. *p < 0.05 vs. adjacent normal tissues. **D** SNAI2 protein expression in glioma tissues and adjacent normal tissues as determined by Western blot assay, *n* = 80. *p < 0.05 vs. adjacent normal tissues. **E** SNAI2 expression in glioma tissues and adjacent normal tissues as determined by immunohistochemistry. **F** Immunohistochemistry score of SNAI2 in glioma tissues and adjacent normal tissues. *n* = 80. **G** Kaplan–Meier analysis for the correlation between SNAI2 expression and OS of patients with glioma.

Previous literature has highlighted the oncogenic transcriptional repressor role of SNAI2 in the development of glioma [9]. We initially evaluated the expression of SNAI2 in glioma datasets GSE50161 and GSE15824 downloaded from the Gene Expression Omnibus database (https://www.ncbi.nlm.nih.gov/gds) as well as the Cancer Genome Atlas (TCGA) database. Predictions suggested that SNAI2 was highly expressed in glioma (Fig. 1A, B). We subsequently determined the expression of SNAI2 in 80 glioma tissues as well as 80 adjacent normal tissues. The RT-qPCR and Western blot assay results revealed that the expression of SNAI2 in the glioma tissues was significantly higher than in that of the normal adjacent normal tissues (*p* < 0.05) (Fig. 1C, D). Moreover, immunohistochemistry results demonstrated that the protein expression of SNAI2 in glioma was markedly higher than that in the normal adjacent normal tissues, and SNAI2 was predominantly distributed in the nuclei (Fig. 1E, F). The Kaplan–Meier method was performed to analyze the correlation between the expression of SNAI2 and the OS of patients with glioma. The results indicated that the OS of patients with high SNAI2 expression was notably lower than that of the patients with low expression of SNAI2, suggesting that high expression of SNAI2 was related to poor prognosis of patients with glioma (Supplementary Table 3) (Fig. 1G). The aforementioned results indicated that SNAI2 was highly expressed in glioma tissues, and that high SNAI2 expression may be associated with poor prognosis in patients with glioma.

### SNAI2 is upregulated in GSCs

**Fig. 2:**
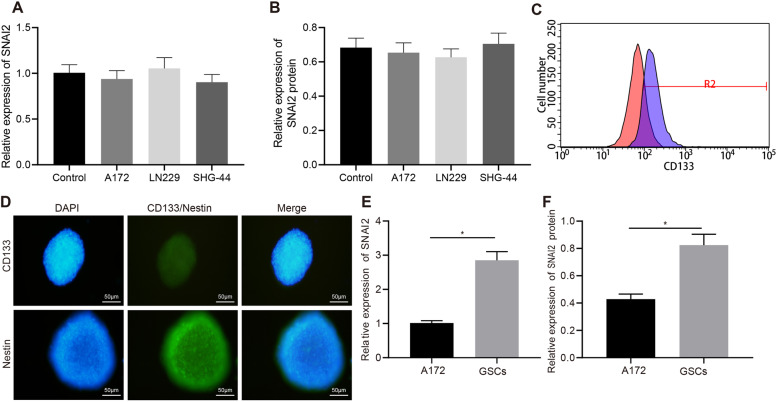
SNAI2 expression is upregulated in GSCs. **A** SNAI2 mRNA expression in A172, LN229, and SHG-44 cells and human astrocytes as determined by RT-qPCR. **B** SNAI2 protein expression in A172, LN229, SHG-44 cells and human astrocytes as determined by Western blot assay. **C** CD133-positive cells as selected by flow cytometry. **D** The expression of CD133 (green) and Nestin (green) as measured by immunofluorescence. DAPI was stained in blue. **E** The mRNA expression of SNAI2 in GSCs and A172 cells as determined by RT-qPCR. **F** The protein expression of SNAI2 in GSCs and A172 cells as determined by Western blot assay. *p < 0.05 vs. A172 cells. Each cell experiment was repeated three times independently.

Next, to further determine the expression of SNAI2 in glioma cells, we collected glioma cell lines A172, LN229, and SHG-44 and selected human astrocytes as the control. No significant difference was detected in terms of the expression of SNAI2 between the different glioma cell lines as determined by RT-qPCR and Western blot assay (Fig. 2A, B). Flow cytometry was subsequently performed to sort out CD133-positive cells from A172 glioma cells (GSCs). The purity of GSCs was examined to be high based on the flow cytometry findings (Fig. 2C). At the same time, immunofluorescence revealed high expression of surface marker proteins of GSCs (CD133 and Nestin), which further suggested that the cells we collected were indeed GSCs (Fig. 2D). The mRNA and protein expression of SNAI2 in GSCs was markedly higher than that in A172 glioma cell line relative to the human astrocytes, which was highlighted by the RT-qPCR and Western blot assay findings (Fig. 2E, F). The aforementioned findings suggest that the expression of SNAI2 was higher in tumor stem cells and SNAI2 may play a role in GSCs rather than non-stem tumor cells.

### SNAI2 promotes the proliferation of GSCs

**Fig. 3:**
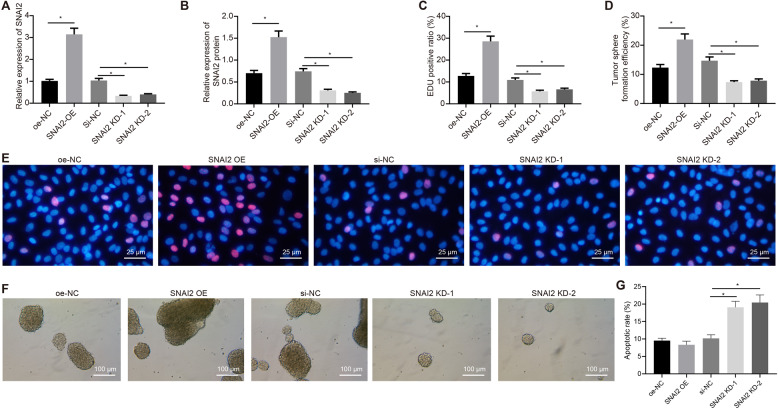
SNAI2 promotes the proliferation of GSCs. **A** The overexpression and knockdown efficiency of SNAI2 in GSCs validated with RT-qPCR. **B** The overexpression and knockdown efficiency of SNAI2 in GSCs as detected by Western blot assay. **C** Statistics of EdU-positive cells in GSCs. **D** Statistics of cell sphere formation efficiency of GSCs. **E** The proliferation of GSCs in response to oe-NC, oe-SNAI2, si-NC or SNAI2-KD as detected by EdU assay. **F** The sphere-forming ability of GSCs in response to oe-NC, oe-SNAI2, si-NC or SNAI2-KD as examined by spheroid formation assay. **G** the apoptosis of GSCs in response to oe-NC, oe-SNAI2, si-NC, or SNAI2-KD as examined by flow cytometry. *p < 0.05 vs. GSCs treated with oe-NC or si-NC. Each cell experiment was repeated three times independently.

Next, to ascertain the biological function of SNAI2 in GSCs, we first constructed the overexpression and knockdown plasmids of SNAI2. Through RT-qPCR and Western blot assay, we found that overexpression of SNAI2 led to an upregulation in the expression of SNAI2 expression, while SNAI2-KD downregulated its expression, validating the successful transfection efficiency of SNAI2 (Figs. 3A, B and S1A). As depicted by the EdU assay, the viability of GSCs was significantly elevated in the presence of oe-SNAI2 compared with oe-NC, while SNAI2-KD triggered a reduction in the viability of GSCs relative to si-NC (*p* < 0.05) (Fig. 3C, E). Additionally, spheroid formation assay demonstrated that oe-SNAI2 contributed to enhanced sphere-forming ability compared with oe-NC and reduced sphere-forming ability was observed in response to SNAI2-KD compared with si-NC (*p* < 0.05) (Fig. 3D, F). Flow cytometry was performed to examine the apoptosis of the GSCs. The results illustrated that oe-SNAI2 did not affect apoptosis, while SNAI2-KD led to a significant elevation in the apoptosis of GSCs relative to si-NC (*p* < 0.05) (Figs. 3G and S2A). The above results demonstrate that SNAI2 promoted the proliferation of GSCs, while an opposite trend was observed following its knockdown.

### Transcription factor SNAI2 activates the Akt pathway by inhibiting PHLPP2

**Fig. 4:**
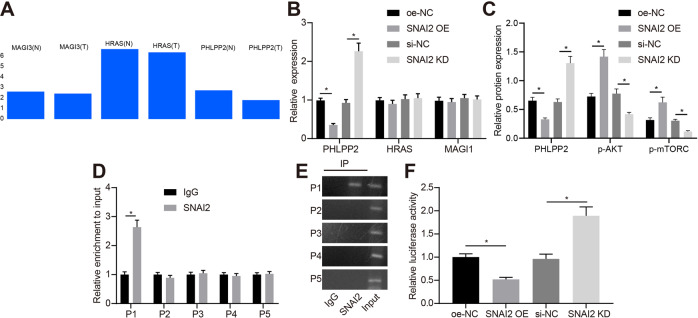
SNAI2 activates the Akt pathway by inhibiting PHLPP2. **A** The expression of MAGI3, HRAS, and PHLPP2 in TCGA, the *x*-axis indicates the sample type and gene name, and the *y*-axis indicates the expression value (N indicates normal samples, T indicates tumor samples). **B** The expression of PHLPP2, MAGI1, and HRAS in the presence of oe-SNAI2 or SNAI2-KD as measured by RT-qPCR. **C** The expression of PHLPP2, p-Akt level, p-mTORC level and mTORC protein expression in the presence of oe-SNAI2 or SNAI2-KD in GSCs as measured by Western blot assay. **D** The binding site between SNAI2 and PHLPP2 promoter as detected by ChIP. **E** The binding site between SNAI2 and PHLPP2 promoter as detected by DNA agarose gel electrophoresis. **F** The binding efficiency between SNAI2 and PHLPP2 promoter as examined by luciferase reporter gene assay. *p < 0.05 vs. GSCs treated with oe-NC or si-NC. Each cell experiment was repeated three times independently.

Further analysis into the function of SNAI2 revealed that SNAI2 was functional as a transcriptional inhibitor [19]. We subsequently set out to further elucidate the mechanism underlying the regulatory role of SNAI2 in biological behaviors of GSCs. The downstream regulatory genes of SNAI2 were predicted using the hTFtarget database (http://bioinfo.life.hust.edu.cn/hTFtarget#!/), which revealed 136 potential regulatory target genes (Supplementary Table 4). KEGG enrichment analysis on the KOBAS 3.0 database (http://kobas.cbi.pku.edu.cn/kobas3/?t=1) illustrated that membrane-associated guanylate kinase with an inverted repeat member 1 (MAGI1), Harvey ras (HRAS) and PHLPP2 were enriched in the PI3K/Akt pathway. By searching for the expression of these three genes in TCGA glioma dataset (http://gepia2.cancer-pku.cn/#index), we uncovered that PHLPP2 exhibited the greatest downregulation (Fig. 4A). These results suggested that SNAI2 regulated the Akt pathway through PHLPP2 to affect the development of glioma.

Next, to verify the aforementioned prediction results, RT-qPCR was performed following the overexpression or knockdown of SNAI2 in the cells, with the results indicating that the expression of PHLPP2 was markedly decreased in response to oe-SNAI2 relative to oe-NC, while a significant increase was detected in relation to SNAI2-KD relative to si-NC (*p* < 0.05); there was no significant change regarding the expression of MAGI1 and HRAS after treatment of either oe-SNAI2 or SNAI2-KD (Fig. 4B). We subsequently examined the activation of Akt pathway as well as the expression of mTORC protein. Our data indicated that GSCs treated with oe-SNAI2 led to a reduction in the expression of PHLPP2, accompanied by elevated p-Akt and p-mTORC levels and increased mTORC protein expression compared with oe-NC. When compared to si-NC, SNAI2-KD treated GSCs contributed to an increase in the expression of PHLPP2, accompanied with a decline in the p-Akt and p-mTORC levels and a decrease in mTORC protein expression (*p* < 0.05) (Figs. 4C and S1B).

Furthermore, our bioinformatics prediction using the JASPAR database (http://jaspar.genereg.net/) revealed five possible sites (P1, P2, P3, P4, P5) in the promoter region of PHLPP2 that may potentially bind to SNAI2 (Supplementary Table 5). ChIP, qPCR and DNA agarose detection experiments provided evidence validating the presence of a binding site of SNAI2 and the PHLPP2 promoter in the P1 region (Fig. 4D, E). Finally, we detected that oe-SNAI2 significantly inhibited the activity of PHLPP2 promoter, while an opposite trend was observed by SNAI2-KD (Fig. 4F). Taken together, the above data indicates that the transcription factor SNAI2 further activates the Akt pathway by downregulating PHLPP2.

### SNAI2 promotes the proliferation of GSCs by inhibiting PHLPP2 to activate the Akt pathway

**Fig. 5:**
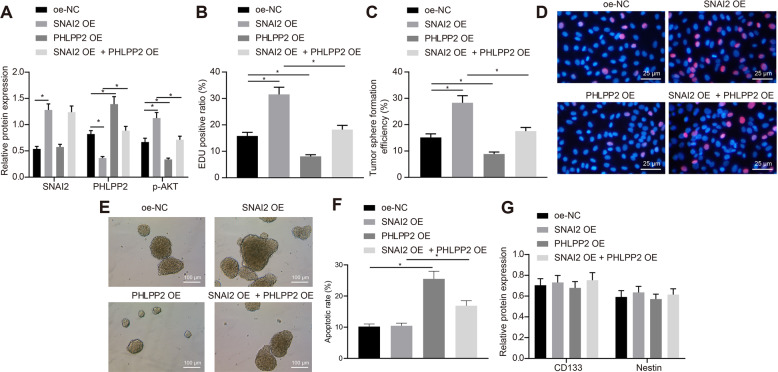
SNAI2 promotes the proliferation of GSCs by inhibiting PHLPP2 to activate the Akt pathway. **A** The p-Akt and PHLPP2 level in the presence of oe-NC, oe-SNAI2, oe-PHLPP2, and oe-SNAI2 + oe-PHLPP2 as determined by Western blot assay. **B** Statistics of EdU-positive cells in GSCs in the presence of oe-NC, oe-SNAI2, oe-PHLPP2, and oe-SNAI2 + oe-PHLPP2 as determined by EdU assay. **C** Statistics of cell sphere formation efficiency of GSCs in the presence of oe-NC, oe-SNAI2, oe-PHLPP2, and oe-SNAI2 + oe-PHLPP2 as examined by spheroid formation assay. **D** Representative images of EdU-positive cells in GSCs. **E** Representative images of sphere formation in GSCs. **F** The apoptotic rate of GSCs in the presence of oe-NC, oe-SNAI2, oe-PHLPP2, and oe-SNAI2 + oe-PHLPP2 as examined by flow cytometry. **G** The expression of CD133 and Nestin on the surface of GSCs in the presence of oe-NC, oe-SNAI2, oe-PHLPP2, and oe-SNAI2 + oe-PHLPP2 as examined by immunofluorescence. *p < 0.05 vs. GSCs treated with oe-NC, si-NC, oe-SNAI2, or oe-PHLPP2. Each cell experiment was repeated three times independently.

To verify the function of the transcription factor SNAI2 activation of the Akt signaling pathway by inhibiting PHLPP2 in GSCs, we constructed an overexpression plasmid of PHLPP2. Western blot assay was performed and the results revealed that the p-Akt level was increased, while the expression of PHLPP2 was decreased by oe-SNAI2 when compared with oe-NC with an opposite trend observed in response to oe-PHLPP2. Overexpressed PHLPP2 was found to reverse the effects of oe-SNAI2 on PHLPP2 and p-Akt level (Figs. 5A and S1C). As shown in the EdU assay, the viability of the GSCs was markedly enhanced in the presence of oe-SNAI2, and reduced by oe-PHLPP2. In comparison to oe-SNAI2, oe-SNAI2 + oe-PHLPP2 notably decreased the viability of GSCs (*p* < 0.05) (Fig. 5B, D). Meanwhile, the sphere-forming ability of the GSCs was increased following treatment with oe-SNAI2, while it was decreased in response to oe-PHLPP2; relative to oe-SNAI2, oe-SNAI2 + oe-PHLPP2 led to reduced sphere-forming ability of GSCs (*p* < 0.05) (Fig. 5C, E). The apoptotic rate of the GSCs was diminished in the presence of oe-SNAI2 but elevated following oe-PHLPP2; GSCs treated with oe-SNAI2 + oe-PHLPP2 exhibited an increased rate of apoptosis in comparison to those treated with oe-SNAI2 (*p* < 0.05) (Figs. 5F and S2B). Our data indicated that the stemness markers CD133 and nestin were not influenced by any of the above treatment protocols (Fig. 5G). Overall, SNAI2 promoted the proliferation of GSCs via activation of the Akt pathway by downregulating dPHLPP2.

### SNAI2 promotes in vivo tumorigenesis of glioma to activate the Akt pathway by inhibiting PHLPP2

**Fig. 6:**
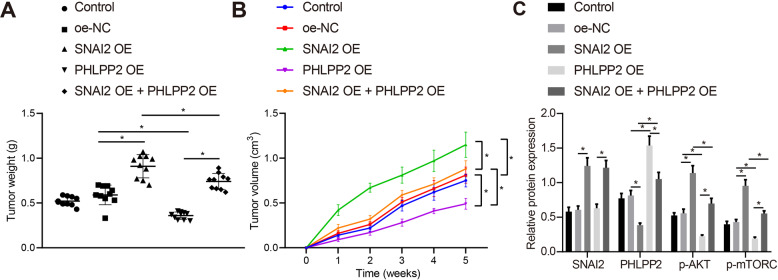
SNAI2 promotes in vivo tumorigenesis of glioma by inhibiting the PHLPP2 to activate the Akt pathway. **A** The weight of tumor xenografted in nude mice in the presence of oe-NC, oe-SNAI2, oe-PHLPP2, or oe-SNAI2 + oe-PHLPP2. **B** The volume of tumor xenografted in nude mice in the presence of oe-NC, oe-SNAI2, oe-PHLPP2, or oe-SNAI2 + oe-PHLPP2. **C** p-Akt level in the presence of oe-NC, oe-SNAI2, oe-PHLPP2, or oe-SNAI2 + oe-PHLPP2 in nude mice xenografted with tumor as determined by Western blot assay. *p < 0.05 vs. nude mice xenografted with tumor injected with GSCs expressing oe-NC, si-NC, oe-SNAI2, or oe-PHLPP2. Each cell experiment was repeated three times independently. *n* = 10.

A xenograft tumor model was constructed in nude mice to further explore the mechanism by which SNAI2 acts to activate the Akt pathway by inhibiting PHLPP2 in vivo. GSCs transfected with oe-NC, oe-SNAI2, oe-PHLPP2, or oe-SNAI2 + oe-PHLPP2 plasmids were subcutaneously injected into the nude mice, with the tumor volume measured on a weekly basis. At the end of the 5-week experiment, the tumor tissues were photographed and weighed. The results revealed that compared with oe-NC, the tumor weight and volume were significantly elevated in response to oe-SNAI2, but were decreased following oe-PHLPP2. GSCs transfected with oe-SNAI2 + oe-PHLPP2 led to marked reduction in tumor weight and volume relative to oe-SNAI2 and increased tumor weight and volume relative to oe-PHLPP2 in nude mice xenografted with tumor (Figs. 6A, B and S3A). The Western blot assay revealed that the p-Akt level was elevated in the presence of oe-SNAI2 but decreased in the presence of oe-PHLPP2. GSCs transfected with oe-SNAI2 + oe-PHLPP2 led to notably decreased p-Akt level relative to oe-SNAI2 and increased p-Akt level relative to oe-PHLPP2 in nude mice xenografted with tumor (Fig. 6C). The H&E staining results revealed there to be no tissue injury after treatment with SNAI2. However, in the presence of oe-PHLPP2, the tissues exhibited distinctive signs of necrosis. Moreover, oe-SNAI2 + oe-PHLPP2 resulted in alleviated tissue injury when compared to oe-PHLPP2 in nude mice xenografted with tumor (Fig. S3B). Furthermore, the p-mTORC and p-Akt expression increased upon oe-SNAI2 but decreased following oe-PHLPP2 when compared to oe-NC in nude mice xenografted with tumor. Relative to oe-SNAI2, oe-SNAI2 + oe-PHLPP2 brought about a decrease in p-mTORC and p-Akt expression; relative to oe-PHLPP2, oe-SNAI2 + oe-PHLPP2 led to an increase in p-mTORC and p-Akt expression (Fig. S3C). Overall, the role of SNAI2 in GSCs through the PHLPP2/Akt axis was successfully verified in our in vivo animal model.

## Discussion

Glioma represents a common intracranial malignant carcinoma well known for its fast growth, frequent recurrence and unsatisfactory prognosis [20]. The transcription factor, SNAI2 is a notable regulator in various tumor cell biological processes, exerting its influence through transcriptional regulation and contributing to disease progression [21]. In the current study, we set out to elucidate the role of SNAI2 in the progression of glioma while exploring the relevant underlying mechanism. Our data validated the initial hypothesis that SNAI2 was an oncogene for glioma through activation of the Akt pathway by downregulating PHLPP2 expression.

Initially, our results revealed that SNAI2 is upregulated in glioma tumors, which was found to be negatively correlated with the OS of patients with glioma. Previous reports have highlighted the upregulation of SNAI2 in glioblastoma tissues when compared with that in non-neoplastic white matter, which is consistent with the observations of our study [22]. Moreover, a previous study indicated that the expression of SNAI2 was elevated in imatinib-resistant glioblastoma cells and was higher in patients with glioblastoma than in those with relapsed glioblastoma [23]. Studies have indicated that when upregulated in a sequential manner with lose-dose BIX01294 treatment, SNAI2 aids in promoting the migration and metastasis of glioblastoma cells [24]. Notably, upregulated SNAI2 has been previously implicated in the self-renewal of cancer stem cells [25]. Consistent with our observations, Xia et. al. emphasized the involvement of SNAI2 in glioma cell stem-like properties; A decrease in SNAI2 elicited by overexpressed miR-124 contributed to a reduction in the CD133(+) cell subpopulation and decreased Nestin expression, while suppressing the in vivo tumorigenicity and invasion of glioma cells [26]. All these previous studies lend support to our findings indicating that SNAI2 promotes the proliferation of GSCs in vitro and facilitated glioma tumor growth in vivo.

Mechanistically, dual luciferase reporter gene assay combined with RT-qPCR and Western blot assay unveiled that SNAI2 trans-suppressed the expression of PHLPP2, which in turn activated the Akt pathway (reflected by increased p-Akt level and mTORC protein expression). As previously reported, the expression of SNAI2 was revealed to share positive association with histopathological grade and survival time of patients with glioma, which was involved with the activation of the PI3K/Akt pathway [27]. Furthermore, SNAI2-knockdown was also reported to suppress the activation of the PI3K/Akt/mTOR pathway, thereby suppressing the development of melanoma [28]. Similarly, SLUG could bind to PTEN promoter region to transcriptionally repress PTEN, thereby regulating the PTEN/AKT pathway and thus possibly favoring prostate cancer stem cell progression [29]. Notably, our study found that SNAI2 could bind to the promoter of PHLPP2, and it was through this less known mechanism that SNAI2 activates the Akt pathway. The involvement of the Akt pathway in GSCs has been largely reported. For instance, the activation of the PI3K/Akt pathway via CD133-p85 interaction was found to enhance tumorigenic ability of GSCs [30]. miR-200b-targeted Akt pathway suppressed the stemness properties as well as division of the CD133(+) glioma cells [31]. Moreover, inhibition of Akt-EphA2 cross-talk suppressed glioblastoma stem cell properties [32]. Intriguingly, PHLPP can exert regulation on Akt inactivation by regulating mTORC expression [33]. PHLPP2 protein phosphatase can inactivate Akt kinase, thereby antagonizing the activity of mTORC2 [34]. Over the past decade, an increasing number or studies have reported the negative regulation by PHLPP2 on the Akt pathway in glioma cells. PHLPP2 phosphatase plays an inhibitory role for glioblastoma cell progression by dephosphorylating and inactivating Akt [35]. Additionally, a reverse relationship between the expression of PHLPP and the p-Akt level was identified in PTEN-negative glioblastoma cells [36]. It was reported that miR-372-downregulated PHLPP2 markedly inhibited cell proliferation while suppressing glioma tumor growth in xenograft mouse model by increasing the phosphorylation levels of Akt and mTOR [37]. Overall, our study discovered the oncogenic role of SNAI2 in glioma was achieved by PHLPP2 downregulation-activated Akt pathway.

**Fig. 7:**
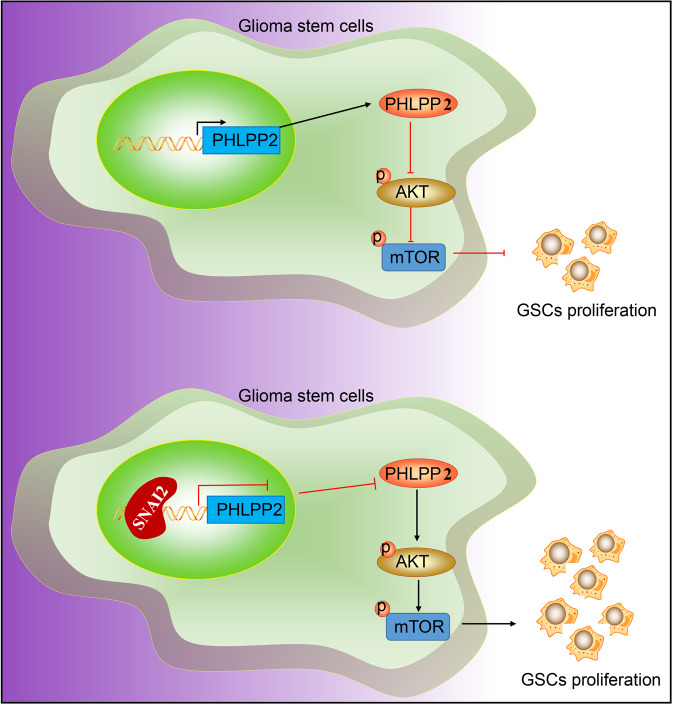
Molecular mechanism regarding the role of SNAI2 in GSCs via the Akt pathway by regulating PHLPP2 in GSC proliferation. SNAI2 downregulates PHLPP2 to activate the Akt pathway, thereby promoting the proliferation of GSCs.

Our results demonstrate that SNAI2 downregulates PHLPP2 to activate the Akt pathway, thereby promoting the proliferation of GSCs (Fig. 7). Our study provides insight into the potential of anti-SNAI2-targeted therapy for glioma treatment, but requires further study and future validation.

## Supplementary information

Supplemental materials

aj-checklist

## Data Availability

The original contributions presented in the study are included in the article/Supplementary Material, further inquiries can be directed to the corresponding author.
