# Supplementary material for: Transcription factor SNAI2 exerts pro-tumorigenic effects on glioma stem cells via PHLPP2-mediated Akt pathway
Source: Cell Death Dis. 2022 Jun 2;13(6):516. doi: 10.1038/s41419-021-04481-2 (PMC9163135; doi:10.1038/s41419-021-04481-2)
Supplement: Supplementary file 1 — Supplemental materials [file 41419_2021_4481_MOESM1_ESM.pdf]

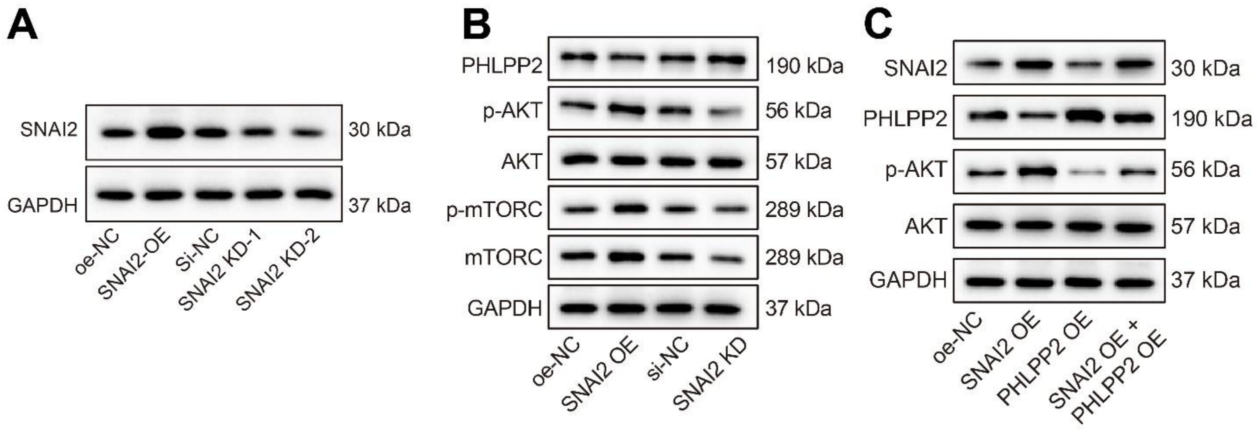

Fig. S1. Representative Western blot images of proteins. A, Representative Western blot images showing overexpression and knockdown efficiency of SNAI2 in GSCs. B, Representative Western blot images of PHLPP2, p-Akt level, p-mTORC, and mTORC proteins in the presence of oe-SNAI2 or SNAI2-KD in GSCs. C, Representative Western blot images of p-Akt and PHLPP2 proteins in the presence of oe-NC, oe-SNAI2, oe-PHLPP2, and oe-SNAI2 + oe-PHLPP2.

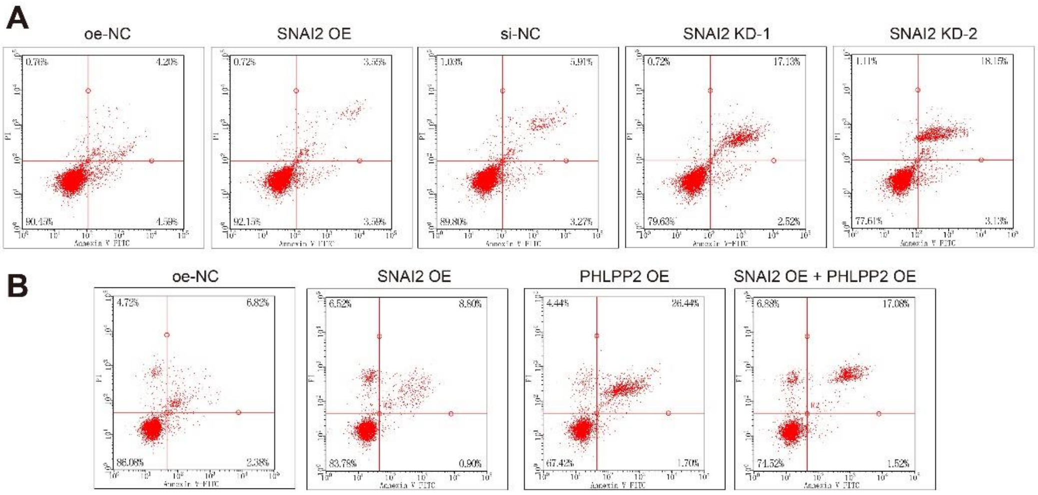

Fig. 2. Flow cytometry scatter plots. A, Flow cytometry scatter plots of apoptotic GSCs in response to oe-NC, oe-SNAI2, si-NC or SNAI2-KD. B, Flow cytometry scatter plots of apoptotic GSCs in the presence of oe-NC, oe-SNAI2, oe-PHLPP2, and oe-SNAI2 + oe-PHLPP2.

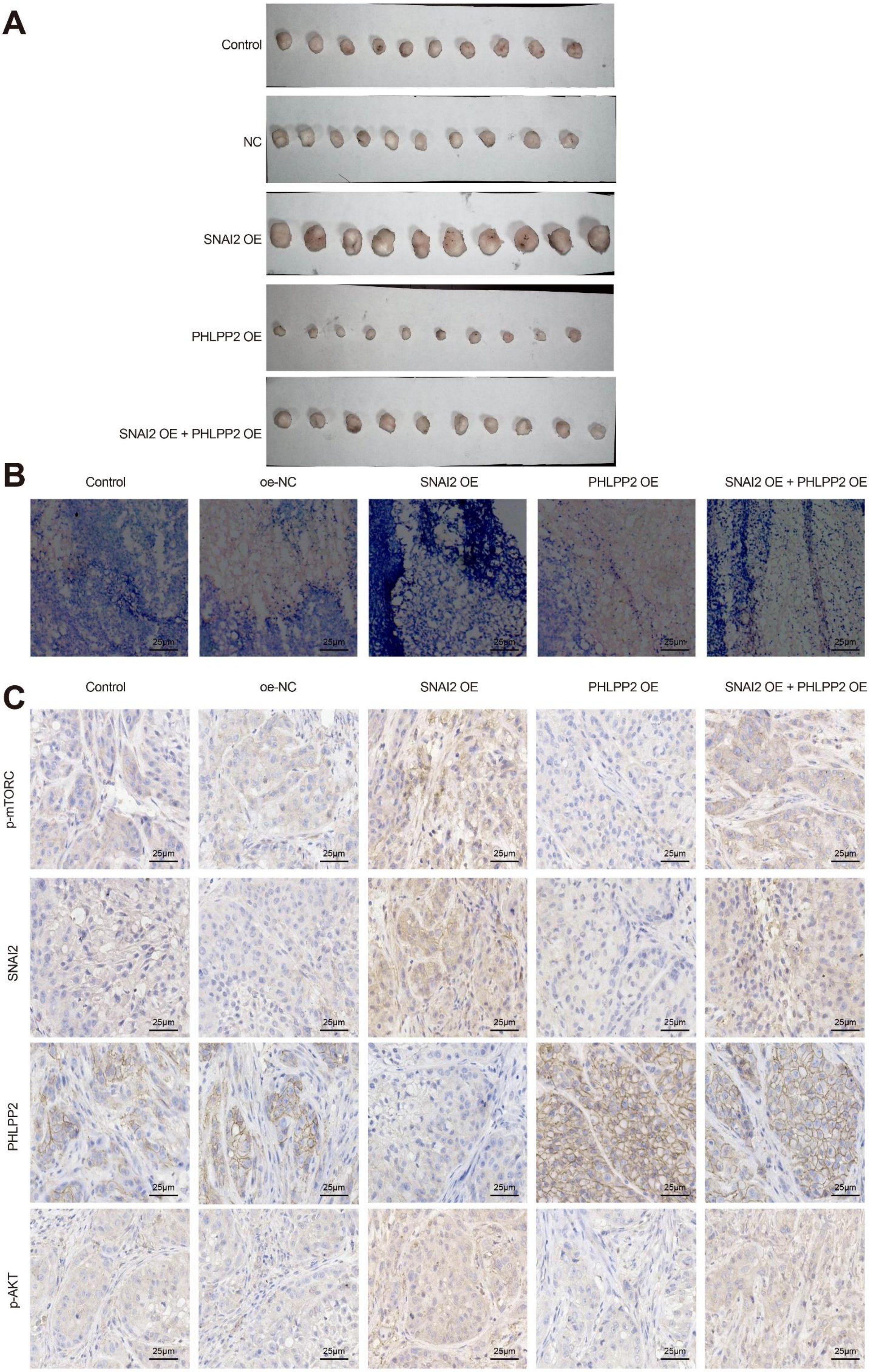
A
Control
NC
SNAI2 OE
PHLPP2 OE
SNAI2 OE + PHLPP2 OE
B
Control
oe-NC
SNAI2 OE
PHLPP2 OE
SNAI2 OE + PHLPP2 OE
25μm
C
Control
oe-NC
SNAI2 OE
PHLPP2 OE
SNAI2 OE + PHLPP2 OE
p-mTORC
SNAI2
PHLPP2
p-AKT
25μm

Fig. S3. Representative images of tumors, tissue damage, and proteins in nude mice. A, Representative images of tumors formed in nude mice. B, Representative HE staining images of damage to tumor tissues. C, Representative immunohistochemical images of p-Akt protein in the presence of oe-NC, oe-SNAI2, oe-PHLPP2 or oe-SNAI2 + oe-PHLPP2 in tumor-bearing nude mice.

Table S1 Primer sequences for RT-qPCR

Gene	Primer sequences
SNAI2	F: 5-ATCTGCGGCAAGGCGTTTTCCA-3
	R: 5-GAGCCCTCAGATTTGACCTGTC-3
GAPDH	F: 5-GTGGACCTGACCTGCCGTCT-3
	R: 5-GGAGGAGTGGGTGTCGCTGT-3
PHLPP2	F: 5-AGGTTCCTGAGCATCTCT-3
	R: 5-GTTCAGGCCCTTCAGTTGAG-3
HRAS	F: 5-CTCACCTCTATAGTGGGGTCGT-3
	R: 5-TGAGGAGCGATGACGGAATA-3
MAGI1	F: 5-CTCTGAACACTGTGAGCTCTGGCAGC-3
	R: 5-TTGGTTTGGTGGTGGTCCTTGTTTCC-3

Notes: SNAI2, Snail family transcriptional repressor 2; GAPDH, glyceraldehyde-3-phosphate dehydrogenase; PHLPP2, PH domain leucine-rich repeat protein phosphatase; HRAS, Harvey ras; MAGI1, membrane-associated guanylate kinase with an inverted repeat member 1; RT-qPCR, reverse transcription quantitative polymerase chain reaction; F, forward; R, reverse.

Table S2 Primer sequences of si-RNA.

	Primer sequences
P1	F: 5’-AAACCTTAACAGATGCGTTCAGCAG-3’
	R: 5’-CCACTAATTGGCTAATAAAAACAGA-3’
P2	F: 5’-ACAGTGCAAAAGGATCGTTTTTTTT-3’
	R: 5’-TGTCATTATTTCAAACTTTTCCAGA-3’
P3	F: 5’-TTCGTCTACTGCAAGAGCCAAGTTC-3’
	R: 5’-CTGGGAAGTGCACGCAGATATG-3’
P4	F: 5’-ATAGGGCAATTCATTTTAGCCATCC-3’
	R: 5’-AAGGAACAAATGAAATGCTATTAAT-3’
P5	F: 5’-CTCTGGGCTGTGAGTGTGAGCGGAC-3’
	R: 5’-TTTGGTGTTTTTGGTCCACTGTGGG-3’
GAPDH	F: 5'-AAGGCTGTGGGCAAGG-3’
	R: 5'-TGGAGGAGTGGGTGTCG-3'

Notes: si-RNA, small interfering RNA; F, forward; R, reverse; GAPDH, glyceraldehyde-3-phosphate dehydrogenase.

Table S3 The clinical data of and the expression of SNAI2 in 80 patients with glioma

Variable	N	SNAI2 expression		*p* Value
		Low	High	
All cases	80	40	40	
Age (years)				0.8217
< 55	35	17	18	
> = 55	45	23	22	
Gender				0.499
Male	45	24	21	
Female	35	16	19	
TNM stage				0.4854
I-II	51	27	24	
III-IV	29	13	16	
Histological grade				0.0139
Low	39	25	14	
High	41	15	26	
Tumor size (cm)				0.6543
< 4	38	18	20	
> 4	42	22	20	

Note: SNAI2, Snail family transcriptional repressor 2; TNM, tumor-node-metastasis; N, number.

Table S4 Potential regulatory target genes by SNAI2

Index	TFs	Gene id	Gene name	Chromosome	Start	End
1	SNAI2	ENSG00000117298	ECE1	Chr1	21217247	21345504
2	SNAI2	ENSG00000203880	PCMTD2	Chr20	64255695	64287821
3	SNAI2	ENSG00000260293	RP11-715J22.6	Chr16	2476558	2482173
4	SNAI2	ENSG00000105339	DENND3	Chr8	141117278	141195808
5	SNAI2	ENSG00000162065	TBC1D24	Chr16	2475146	2505734
6	SNAI2	ENSG00000235194	PPP1R3E	Chr14	23295643	23302848
7	SNAI2	ENSG00000117984	CTSD	Chr11	1752752	1763992
8	SNAI2	ENSG00000103855	CD276	Chr15	73683966	73714518
9	SNAI2	ENSG00000163399	ATP1A1	Chr1	116372668	116410261
10	SNAI2	ENSG00000155034	FBXL18	Chr7	5431335	5513798
11	SNAI2	ENSG00000009950	MLXIPL	Chr7	73593194	73624543
12	SNAI2	ENSG00000159267	HLCS	Chr21	36750888	36990236
13	SNAI2	ENSG00000178057	NDUFAF3	Chr3	49020459	49023495
14	SNAI2	ENSG00000103184	SEC14L5	Chr16	4958317	5019158
15	SNAI2	ENSG00000273001	RP11-118K6.3	Chr10	3065424	3066001
16	SNAI2	ENSG00000239930	AP001625.4	Chr21	42496539	42497443
17	SNAI2	ENSG00000207613	MIR181C	Chr19	13874699	13874808
18	SNAI2	ENSG00000270259	RP13-122B23.9	Chr9	137286112	137287236
19	SNAI2	ENSG00000199032	MIR425	Chr3	49020148	49020234
20	SNAI2	ENSG00000095383	TBC1D2	Chr9	98198999	98255721
21	SNAI2	ENSG00000226334	RP11-217B7.2	Chr9	104927553	104928892
22	SNAI2	ENSG00000150967	ABCB9	Chr12	122920951	122981649
23	SNAI2	ENSG00000040199	PHLPP2	Chr16	71637835	71724701
24	SNAI2	ENSG00000199038	MIR210	Chr11	568089	568198
25	SNAI2	ENSG00000265587	AC068580.1	Chr11	1763931	1764025
26	SNAI2	ENSG00000173846	PLK3	Chr1	44800225	44805990
27	SNAI2	ENSG00000279873	LINC01126	Chr2	43227341	43228855
28	SNAI2	ENSG00000237172	B3GNT9	Chr16	67148105	67151214
29	SNAI2	ENSG00000213801	ZNF816-ZNF321P	Chr19	52927135	52942601
30	SNAI2	ENSG00000034677	RNF19A	Chr8	100257059	100410015
31	SNAI2	ENSG00000267395	AC074212.6	Chr19	45767796	45772504
32	SNAI2	ENSG00000152518	ZFP36L2	Chr2	43222402	43226609
33	SNAI2	ENSG00000196366	C9orf163	Chr9	136483495	136486067
34	SNAI2	ENSG00000241973	PI4KA	Chr22	20707691	20859417
35	SNAI2	ENSG00000100151	PICK1	Chr22	38056311	38075701
36	SNAI2	ENSG00000102871	TRADD	Chr16	67154180	67160298
37	SNAI2	ENSG00000207585	MIR181D	Chr19	13874875	13875011
38	SNAI2	ENSG00000102878	HSF4	Chr16	67163385	67169945
39	SNAI2	ENSG00000187556	NANOS3	Chr19	13862063	13880757
40	SNAI2	ENSG00000188483	IER5L	Chr9	129175552	129178262
41	SNAI2	ENSG00000170542	SERPINB9	Chr6	2887266	2903280
42	SNAI2	ENSG00000181031	RPH3AL	Chr17	212389	386254
43	SNAI2	ENSG00000179627	ZBTB42	Chr14	104800596	104804712
44	SNAI2	ENSG00000178035	IMPDH2	Chr3	49024325	49029408

45	SNAI2	ENSG00000198435	NRARP	Chr9	137300482	137302251
46	SNAI2	ENSG00000174775	HRAS	Chr11	532242	537287
47	SNAI2	ENSG00000128536	CDHR3	Chr7	105876796	106033773
48	SNAI2	ENSG00000173611	SCAI	Chr9	124942608	125143506
49	SNAI2	ENSG00000107263	RAPGEF1	Chr9	131576770	131740074
50	SNAI2	ENSG00000134864	GGACT	Chr13	100530164	100589528
51	SNAI2	ENSG00000142959	BEST4	Chr1	44783585	44787705
52	SNAI2	ENSG00000067057	PFKP	Chr10	3066333	3137712
53	SNAI2	ENSG00000165029	ABCA1	Chr9	104781002	104928237
54	SNAI2	ENSG00000265529	AL031721.1	Chr16	1379289	1379423
55	SNAI2	ENSG00000118898	PPL	Chr16	4882507	4960741
56	SNAI2	ENSG00000235499	AC073046.25	Chr2	73985132	73986343
57	SNAI2	ENSG00000148396	SEC16A	Chr9	136440096	136483759
58	SNAI2	ENSG00000279181	RP11-711M9.2	Chr2	73998337	73998548
59	SNAI2	ENSG00000204055	RP11-247A12.2	Chr9	129176771	129210548
60	SNAI2	ENSG00000213386	RP11-779O18.2	Chr5	172762521	172763258
61	SNAI2	ENSG00000118257	NRP2	Chr2	205681990	205798133
62	SNAI2	ENSG00000178149	DALRD3	Chr3	49015488	49022293
63	SNAI2	ENSG00000187605	TET3	Chr2	73986404	74108176
64	SNAI2	ENSG00000100307	CBX7	Chr22	39120167	39152674
65	SNAI2	ENSG00000187609	EXD3	Chr9	137306896	137423262
66	SNAI2	ENSG00000076706	MCAM	Chr11	119308529	119321521
67	SNAI2	ENSG00000265690	RP11-5A19.5	Chr16	67163385	67165815
68	SNAI2	ENSG00000106635	BCL7B	Chr7	73536356	73558002
69	SNAI2	ENSG00000261513	RP11-432I5.8	Chr16	71723180	71724230
70	SNAI2	ENSG00000059145	UNKL	Chr16	1363205	1414751
71	SNAI2	ENSG00000220785	MTMR9LP	Chr1	32231658	32241620
72	SNAI2	ENSG00000239365	RPS26P49	Chr14	104830838	104831171
73	SNAI2	ENSG00000178252	WDR6	Chr3	49007062	49015953
74	SNAI2	ENSG00000176531	PHLDB3	Chr19	43474954	43504935
75	SNAI2	ENSG00000148384	INPP5E	Chr9	136428619	136439822
76	SNAI2	ENSG00000251161	RP11-540O11.1	Chr15	40906811	40910337
77	SNAI2	ENSG00000229512	AC068580.5	Chr11	1763009	1763749
78	SNAI2	ENSG00000228719	RP5-1119A7.14	Chr22	36445395	36454944
79	SNAI2	ENSG00000268120	CTD-3193O13.11	Chr19	7870561	7871296
80	SNAI2	ENSG00000115266	APC2	Chr19	1446302	1473244
81	SNAI2	ENSG00000260996	RP13-122B23.8	Chr9	137293868	137295721
82	SNAI2	ENSG00000161328	LRRC56	Chr11	537527	554916
83	SNAI2	ENSG00000151276	MAGI1	Chr3	65353525	66038834
84	SNAI2	ENSG00000133069	TMCC2	Chr1	205228176	205273343
85	SNAI2	ENSG00000182253	SYNM	Chr15	99098217	99135593
86	SNAI2	ENSG00000018408	WWTR1	Chr3	149517235	149736714
87	SNAI2	ENSG00000104936	DMPK	Chr19	45769717	45782552
88	SNAI2	ENSG00000109654	TRIM2	Chr4	153152342	153339320
89	SNAI2	ENSG00000252225	Y_RNA	Chr22	36440880	36440970
90	SNAI2	ENSG00000162039	MEIOB	Chr16	1833983	1884294

91	SNAI2	ENSG00000227009	FUNDC2P4	Chr22	39155525	39155945
92	SNAI2	ENSG00000185522	LMNTD2	Chr11	554855	560779
93	SNAI2	ENSG00000131069	ACSS2	Chr20	34872146	34927962
94	SNAI2	ENSG00000183615	FAM167B	Chr1	32247233	32248856
95	SNAI2	ENSG00000177045	SIX5	Chr19	45764785	45769226
96	SNAI2	ENSG00000130222	GADD45G	Chr9	89605013	89606555
97	SNAI2	ENSG00000166925	TSC22D4	Chr7	100463359	100479279
98	SNAI2	ENSG00000131067	GGT7	Chr20	34844720	34872860
99	SNAI2	ENSG00000135127	CCDC64	Chr12	119989869	120094494
100	SNAI2	ENSG00000135722	FBXL8	Chr16	67159931	67164570
101	SNAI2	ENSG00000099849	RASSF7	Chr11	560404	564021
102	SNAI2	ENSG00000231867	AP001625.5	Chr21	42508624	42509661
103	SNAI2	ENSG00000198113	TOR4A	Chr9	137277749	137282641
104	SNAI2	ENSG00000104983	CCDC61	Chr19	45995461	46021318
105	SNAI2	ENSG00000119283	TRIM67	Chr1	231162112	231221556
106	SNAI2	ENSG00000272449	RP3-395M20.12	Chr1	2546465	2547460
107	SNAI2	ENSG00000132016	C19orf57	Chr19	13882348	13906452
108	SNAI2	ENSG00000254815	RP11-496I9.1	Chr11	557595	560107
109	SNAI2	ENSG00000142197	DOPEY2	Chr21	36156782	36294274
110	SNAI2	ENSG00000247095	MIR210HG	Chr11	565660	568457
111	SNAI2	ENSG00000272434	RP13-131K19.6	Chr3	49029316	49029706
112	SNAI2	ENSG00000027075	PRKCH	Chr14	61187559	61550976
113	SNAI2	ENSG00000161021	MAML1	Chr5	179732850	179796511
114	SNAI2	ENSG00000160190	SLC37A1	Chr21	42496008	42581440
115	SNAI2	ENSG00000133805	AMPD3	Chr11	10308313	10507579
116	SNAI2	ENSG00000134531	EMP1	Chr12	13196716	13219939
117	SNAI2	ENSG00000142459	EVI5L	Chr19	7830233	7864976
118	SNAI2	ENSG00000167642	SPINT2	Chr19	38244035	38292614
119	SNAI2	ENSG00000182866	LCK	Chr1	32251239	32286165
120	SNAI2	ENSG00000101298	SNPH	Chr20	1266316	1309328
121	SNAI2	ENSG00000135083	CCNJL	Chr5	160251652	160345396
122	SNAI2	ENSG00000269153	LYPLA2P2	Chr19	7879445	7880120
123	SNAI2	ENSG00000099338	CATSPERG	Chr19	38335775	38370943
124	SNAI2	ENSG00000252575	BX470187.1	Chr22	36438824	36438926
125	SNAI2	ENSG00000237883	DGUOK-AS1	Chr2	73947642	73981441
126	SNAI2	ENSG00000236936	RP3-329E20.2	Chr1	21266082	21267251
127	SNAI2	ENSG00000241313	WWTR1-AS1	Chr3	149657020	149661364
128	SNAI2	ENSG00000099889	ARVCF	Chr22	19969896	20016808
129	SNAI2	ENSG00000160188	RSPH1	Chr21	42472486	42496354
130	SNAI2	ENSG00000236830	CBR3-AS1	Chr21	36131767	36175815
131	SNAI2	ENSG00000099940	SNAP29	Chr22	20858983	20891218
132	SNAI2	ENSG00000183248	FLJ22184	Chr19	7868719	7874379
133	SNAI2	ENSG00000181588	MEX3D	Chr19	1554669	1568058
134	SNAI2	ENSG00000259475	RP11-654A16.3	Chr15	98954149	99105824
135	SNAI2	ENSG00000207605	MIR191	Chr3	49020602	49020714
136	SNAI2	ENSG00000075624	ACTB	Chr7	5527151	5563784

Table S5 Possible binding sites between SNAI2 and PHLPP2 predicted by JASPAR database

Matrix ID	Name	Score	Relative score	Sequence ID	Start	End	Strand	Predicted sequence
MA0745.1	SNAI2	11.9049	0.983983179	PHLPP2	267	275	-	tacaggtgt
MA0745.2	SNAI2	14.2152	0.950717336	PHLPP2	265	277	+	tcacacctgtaat
MA0745.1	SNAI2	9.93734	0.944736193	PHLPP2	876	884	-	aacaagtgt
MA0745.1	SNAI2	9.43432	0.934702239	PHLPP2	468	476	-	tgcaggttc
MA0745.1	SNAI2	9.43432	0.934702239	PHLPP2	1242	1250	-	tgcaggttc
MA0745.1	SNAI2	9.04168	0.926870123	PHLPP2	250	258	+	gccaggtgt
MA0745.1	SNAI2	7.73248	0.900755059	PHLPP2	804	812	+	ctcaggtga
MA0745.1	SNAI2	6.7364	0.880885889	PHLPP2	406	414	-	cacaagtga
MA0745.1	SNAI2	6.71767	0.880512195	PHLPP2	1074	1082	+	aggaggtgg
MA0745.1	SNAI2	6.61007	0.878365877	PHLPP2	658	666	-	gggaggtgg
MA0745.1	SNAI2	6.61007	0.878365877	PHLPP2	1514	1522	-	gggaggtgg
MA0745.1	SNAI2	6.18189	0.869824714	PHLPP2	1723	1731	+	tacaagttg
MA0745.1	SNAI2	5.53447	0.856910465	PHLPP2	571	579	-	aaaaagtgt
MA0745.1	SNAI2	5.42471	0.854721013	PHLPP2	670	678	+	ttcaagtga
MA0745.1	SNAI2	5.39908	0.854209656	PHLPP2	1033	1041	-	gaaaggtac
MA0745.2	SNAI2	8.31855	0.852941939	PHLPP2	248	260	-	ccacacctggctg
MA0745.1	SNAI2	5.14632	0.849167787	PHLPP2	1102	1110	-	ttaaggtgg
MA0745.1	SNAI2	4.95683	0.845387965	PHLPP2	968	976	-	aactggtat
MA0745.2	SNAI2	7.82116	0.844694358	PHLPP2	1534	1546	-	gcacgcctgtaat
MA0745.1	SNAI2	4.53186	0.836911014	PHLPP2	1520	1528	+	cccaagtgg
MA0745.1	SNAI2	4.40581	0.834396491	PHLPP2	1536	1544	+	tacaggcgt
MA0745.1	SNAI2	4.36519	0.833586268	PHLPP2	1204	1212	-	cacaagtta
MA0745.2	SNAI2	6.73877	0.82674673	PHLPP2	466	478	+	ttgaacctgcagt
MA0745.2	SNAI2	6.51774	0.823081654	PHLPP2	874	886	+	aaacacttgttct
MA0745.1	SNAI2	3.76226	0.821559487	PHLPP2	433	441	+	ctgaggtgg
MA0745.1	SNAI2	3.58608	0.818045049	PHLPP2	210	218	+	aaaaggtaa
MA0745.1	SNAI2	3.5435	0.817195748	PHLPP2	460	468	+	tggaggttg
MA0745.1	SNAI2	3.5435	0.817195748	PHLPP2	652	660	-	tggaggttg
MA0745.1	SNAI2	3.48721	0.816072905	PHLPP2	1109	1117	-	gtctggttt
MA0745.1	SNAI2	3.44407	0.815212313	PHLPP2	1989	1997	+	atcaagtaa
MA0745.1	SNAI2	3.37563	0.813847132	PHLPP2	330	338	-	agctggtct
MA0745.1	SNAI2	3.36218	0.813578874	PHLPP2	1054	1062	+	tcaaggtgg
MA0745.1	SNAI2	3.32962	0.812929337	PHLPP2	447	455	-	ctaaggtga
MA0745.1	SNAI2	3.26803	0.811700805	PHLPP2	1610	1618	+	ggctggtct
MA0745.1	SNAI2	3.0808	0.807965982	PHLPP2	1476	1484	-	cggaggttg
MA0745.1	SNAI2	2.91432	0.804645273	PHLPP2	890	898	-	aaaatgtgg
MA0745.1	SNAI2	2.90633	0.804485758	PHLPP2	1937	1945	-	gccaagtag
MA0745.1	SNAI2	2.72725	0.800913641	PHLPP2	664	672	+	cccaagttc
MA0745.1	SNAI2	2.7116	0.800601407	PHLPP2	1683	1691	+	tgaaagtga
